# Cerebral blood flow alterations in migraine patients with and without aura: An arterial spin labeling study

**DOI:** 10.1186/s10194-022-01501-0

**Published:** 2022-10-04

**Authors:** Tong Fu, Lindong Liu, Xiaobin Huang, Di Zhang, Yujia Gao, Xindao Yin, Hai Lin, Yongming Dai, Xinying Wu

**Affiliations:** 1 Department of Radiology, Nanjing First Hospital, Nanjing Medical University, No.68, Changle Road, 210006 Nanjing, Jiangsu Province China; 2 Central Research Institute, United Imaging Healthcare, Shanghai, China

**Keywords:** Migraine with aura, Migraine without aura, Arterial spin labeling, Machine learning approach

## Abstract

**Background:**

Migraine aura is a transient, fully reversible visual, sensory, or other central nervous system symptom that classically precedes migraine headache. This study aimed to investigate cerebral blood flow (CBF) alterations of migraine with aura patients (MwA) and without aura patients (MwoA) during inter-ictal periods, using arterial spin labeling (ASL).

**Methods:**

We evaluated 88 migraine patients (32 MwA) and 44 healthy control subjects (HC) who underwent a three-dimensional pseudo-continuous ASL MRI scanning. Voxel-based comparison of normalized CBF was conducted between MwA and MwoA. The relationship between CBF variation and clinical scale assessment was further analyzed. The mean CBF values in brain regions showed significant differences were calculated and considered as imaging features. Based on these features, different machine learning–based models were established to differentiate MwA and MwoA under five-fold cross validation. The predictive ability of the optimal model was further tested in an independent sample of 30 migraine patients (10 MwA).

**Results:**

In comparison to MwoA and HC, MwA exhibited higher CBF levels in the bilateral superior frontal gyrus, bilateral postcentral gyrus and cerebellum, and lower CBF levels in the bilateral middle frontal gyrus, thalamus and medioventral occipital cortex (all p values < 0.05). These variations were also significantly correlated with multiple clinical rating scales about headache severity, quality of life and emotion. On basis of these CBF features, the accuracies and areas under curve of the final model in the training and testing samples were 84.3% and 0.872, 83.3% and 0.860 in discriminating patients with and without aura, respectively.

**Conclusion:**

In this study, CBF abnormalities of MwA were identified in multiple brain regions, which might help better understand migraine-stroke connection mechanisms and may guide patient-specific decision-making.

## Background

Migraine is a neurovascular disorder and considered as the second leading cause of disability-adjusted life-years lost just after stroke [1]. Patients suffering from migraine are at risk for cryptogenic or cardioembolic stroke, and even a history of migraine is sufficient to accelerate infarct progress and lead to worse consequences [2, 3]. Of note, the risk of stroke in migraine with aura (MwA) is higher than that in migraine without aura (MwoA) [4, 5] Migraine with aura is a complex neurological manifesting clinically as visual, somatosensory, speech, and/or motor symptoms that precedes the headache phase. It may present with acute deficits, mimicking an acute ischemic stroke [5]. Currently, the standard method to subtype migraine patients into MwA and MwoA is following the International Classification of Headache Disorders (ICHD) diagnostic criteria [6], which is relatively subjective and lack of migraine-stroke connection information. To better understand the pathophysiology of migraine-related stroke and choose optimal migraine-specific treatment to avoid potential neurovascular events, non-invasive imaging markers with sufficient reliability in migraine subtyping is desirable.

The pathophysiology mechanism underlying the migraine-stroke connection is still unclear, cortical spreading depolarization and cerebral microembolism that affecting the brain tissue perfusion are the most convincing theories [4]. Functional MRI (fMRI) had been proved to be a useful technology to identify functional connectivity alterations in migraine which is considered being associated with cortical spreading depolarization theories [7, 8]. However, the blood-oxygenation-level-dependent (BOLD) signal derived from fMRI does not directly reflect cerebral perfusion, which makes it difficult to interpret migraine-stroke associated brain activity. Dynamic contrast-enhanced (DCE) MRI, providing direct brain blood flow information, were applied to identify the dysfunction of the blood-brain barrier in migraine which is also highly associated with the brain perfusion [9, 10] However, DCE-MRI on migraine patients should be proceeded with caution and not preferentially recommended [11]. With no contrast agent needed, arterial spin labeling (ASL) technique provides a perfusion assessment without worrying about contrast allergies or renal impairment. It has been conducted to detect cerebral blood flow (CBF) alterations of brain tissue in patients suffering from migraine [12]. However, the results of some previous ASL studies on migraine patients either provided the whole cerebral blood flow and lacked of specificity, did not focus on migraine aura patients or had a small sample size which possibly limited the statistical representation [13–17]. In a fMRI study with 116 MwoA patients, imaging markers of MwoA were identified and validated, which highlighted the role of machine learning methods in identifying MRI biomarkers with high diagnostic value in migraine [18, 19]. The study using a machine learning approach to identify MwA-specific biomarkers that reflect brain tissue perfusion is also needed.

Thus, this study conducted a voxel-based comparison of normalized CBF between MwA and MwoA and applied different machine learning–based models to combine identified CBF features to discriminate MwA from MwoA. The performance of the optimal model constructed from the previous training set would further be evaluated in an independence testing set with the similar ratio of MwA and MwoA patient numbers to the training set.

## Methods

### Participants and clinical assessment

The human study was approved by the local ethics committee, and written informed consent was obtained from each participant. Patients were recruited from the neurological wards. According to the third version of the International Classification of Headache Disorders (ICHD-3) [6], nighty-seven patients were diagnosed as migraine. Diagnostic criteria of migraine without aura and migraine with aura were then used to classify migraine patients into MwoA and MwA groups. Patients with probable migraine, additional neurological disease other than migraine, severe head injury, drug abuse, other major medical illness, brain vascular disease, or hydrocephalus, as well as failing to finish the MR examination were excluded from the study. After screening, 88 migraine patients were finally enrolled into the training sample, including 56 MwoA and 32 MwA (22 of them have visual or retinal symptoms, 8 of them have sensory symptom, 4 of them have speech and/or language symptoms, one of them has motor symptom) patients. 44 healthy control subjects (HC) who were matched to patients in terms of age, sex and education were also enrolled into our study. They were recruited from the local population and had no personal or family history of migraine, or any other types of headaches. To minimize hormonal influences on cortical excitability, all female subjects were included at mid-cycle and excluded if being pregnant or breast-feeding. Migraine patients and HC were all right-handers according to self-report.

All patients completed a neuropsychological assessment including the Self-Rating Anxiety Scale (SAS), Self-Rating Depression Scale (SDS), Montreal Cognitive Assessment (MoCA), Headache Impact Test-6 (HIT-6), and Migraine Disability Assessment Score (MIDAS).

To valid the imaging markers that may be derived from the 88 migraine patients mentioned above an external testing set from the neurological wards of our branch hospital were also recruited. The same inclusion and exclusion criteria of migraine, MwA and MwoA and the same clinical evaluations were used and conducted to enroll patients for testing sample. In final, 30 migraine patients (10 MwA) were included into the testing sample.

### Image acquisition and preprocessing

After at least 4 h fasting, all subjects underwent MR examinations at one of two different 3.0 Tesla MRI scanners (uMR 780, United Imaging Healthcare, Shanghai, China; Ingenia, Philips Medical Systems, Best, Netherlands) for the patients in the training and testing samples, respectively. All subjects were scanned with a protocol including a high-resolution three-dimensional fast-echo T1-weighted MR image (resolution 1 × 1 × 1 mm^3^, TR/TE = 8.1/3.7 mm, slices = 170, FA = 8°, acquisition matrix = 256 × 256, FOV = 256 mm × 256 mm) and a three-dimensional pseudo-continuous ASL image (TR = 4000 ms, label duration = 1650 ms, TE = 11 ms, FA = 90°, post-label delay = 1600 ms, FOV = 240 mm × 240 mm, thickness = 4 mm, gap = 0.4 mm, acquisition matrix = 64 × 64, axial slices = 20). Finally, each subject contained 60 volumes used as 30 label-control image pairs.

The ASL data was preprocessed using the Statistical parameter mapping software (SPM12) (https://www.fil.ion.ucl.ac.uk/spm/software/spm12/) and the toolbox ASLtbx (https://cfn.upenn.edu/~zewan). The procedure for obtaining CBF maps was detailed in our previous study [20]. The major steps included removing skull and cropping the gap, correcting motion artifacts, acquiring frame-wise displacement (FD) between groups and calculating CBF map. The CBF images were linearly co-registered in the native space to their corresponding T1-weighted images, which were non-linearly registered to the standard MNI space (the ICBM152 template). Then, each CBF image underwent spatial smoothing using a Gaussian kernel of FWHM of 8 mm. Afterwards, CBF map was normalized by dividing the value of cerebral blood flow in each voxel (2 mm × 2 mm × 2 mm) with the mean value of the whole brain CBF.

### Voxel- and ROI- based comparisons

The voxel-based comparison of normalized CBF was conducted using a two-sample t-test to identify CBF variations between MwA and MwoA. Statistical threshold was set at t > 3.0 and p < 0.05, false discovery rate (FDR) corrected at cluster level. The brain regions showing significant differences were extracted as ROIs and the mean normalized CBF value in each ROI was calculated as an imaging feature and further pair-wise compared among MwA, MwoA and HC.

### Model construction and evaluation

There were 88 and 30 migraine patients in the training and testing sets, with nearly the same percentage of MwoA and MwA patients (p = 0.76 in a chi-squared test). Based on the identified imaging features from the training set, five types of machine learning–based models including support vector machine (SVM) with the gaussian kernel, k-nearest neighbor, random forest, naive bayes and linear discriminant analysis were established to differentiate MwA and MwoA under 100 runs of five-fold cross validation. The predictive ability of the optimal model was further evaluated in the testing set using a receiver operating characteristic (ROC) curve.

## Results

### Demographic characteristics and clinical assessment of all subjects

The demographic characteristics and clinical assessment of all patients were summarized in Table 1. There were no significant differences in age, gender, education, disease duration, migraine frequency, HIT-6, MIDAS and MoCA score between MwA and MwoA patients, using a chi-squared test for gender and two-tailed t-tests for continuous variables. The MwA group showed higher headache severity score, SAS and SDS scores compared to the MwoA group (all p values < 0.01). Moreover, there were also no significant differences in age, gender, disease duration, migraine frequency, clinical rating scales between patients in the training and testing sets (all p values > 0.05), except for education and MoCA score (both p values < 0.01).

**Table 1:** The demographic and clinical outcome of all patients

	MwA in the training sample (n = 32)	MwoA in the training sample (n = 56)	p-value^a^	Patients in the training sample (n = 88)	Patients in the testing sample (n = 30)	p-value^a^
Age (years)	35.4 ± 12.1	37.0 ± 8.9	0.48	36.4 ± 10.1	37.2 ± 8.6	0.70
Gender (M/F)	7/25	11/45	0.80^b^	18/70	7/23	0.74^b^
Education (years)	13.4 ± 3.7	14.0 ± 3.2	0.48	13.8 ± 3.4	15.5 ± 1.6	< 0.01
Duration (years)	11.8 ± 8.7	14.4 ± 8.8	0.19	13.4 ± 8.8	13.1 ± 8.9	0.87
Frequency (days per month)	4.1 ± 4.0	5.4 ± 6.6	0.35	4.8 ± 5.7	5.2 ± 5.6	0.74
Headache severity score	6.0 ± 1.4	4.3 ± 1.2	< 0.01	5.0 ± 1.5	4.6 ± 1.0	0.18
HIT-6	60.5 ± 7.7	59.6 ± 7.3	0.61	59.9 ± 7.4	61.4 ± 6.3	0.32
MIDAS	19.2 ± 20.0	17.5 ± 21.8	0.75	18.2 ± 20.9	17.2 ± 14.1	0.81
MoCA	25.8 ± 3.1	25.7 ± 3.2	0.97	25.7 ± 3.2	29.6 ± 0.8	< 0.01
SAS	52.4 ± 5.2	43.6 ± 7.0	< 0.01	46.8 ± 9.6	49.0 ± 13.5	0.33
SDS	47.2 ± 7.1	39.0 ± 6.0	< 0.01	42.0 ± 8.7	45.6 ± 12.0	0.08

HIT-6: Headache Impact Test-6, MIDAS: Migraine Disability Assessment Score, MoCA: Montreal Cognitive Assessment, MwA: migraine patients with aura, MwoA: migraine patients without aura, SAS: Self-Rating Anxiety Scale, SDS: Self-Rating Depression Scale

Values are represented as the mean ± standard deviation, except for the gender distribution

^a^Unless otherwise indicated, p values were calculated with two-tailed t-tests

^b^The p values were obtained using chi-squared tests

### The results of voxel- and ROI- based comparisons

**Fig. 1:**
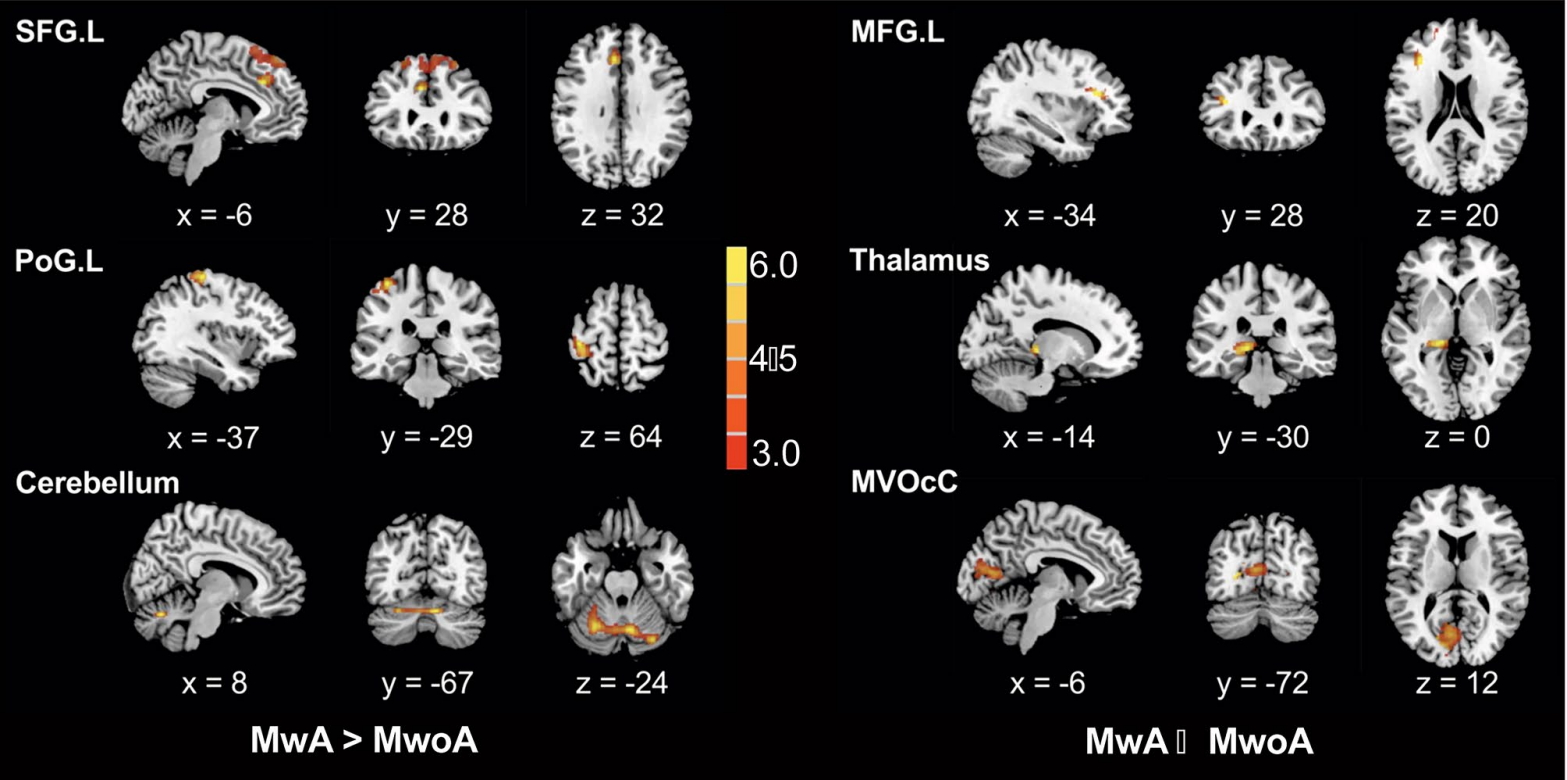
Voxel-based-analysis indicated the brain regions with significant differences in normalized CBF between MwA and MwoA. Statistical threshold was set at t > 3.0 and p < 0.05, FDR corrected at cluster level. CBF: cerebral blood flow, MFG.L: left middle frontal gyrus, MVOcC: medioventral occipital cortex, MwA: migraine patients with aura, MwoA: migraine patients without aura, PoG.L: left postcentral gyrus, SFG.L: left superior frontal gyrus

**Fig. 2:**
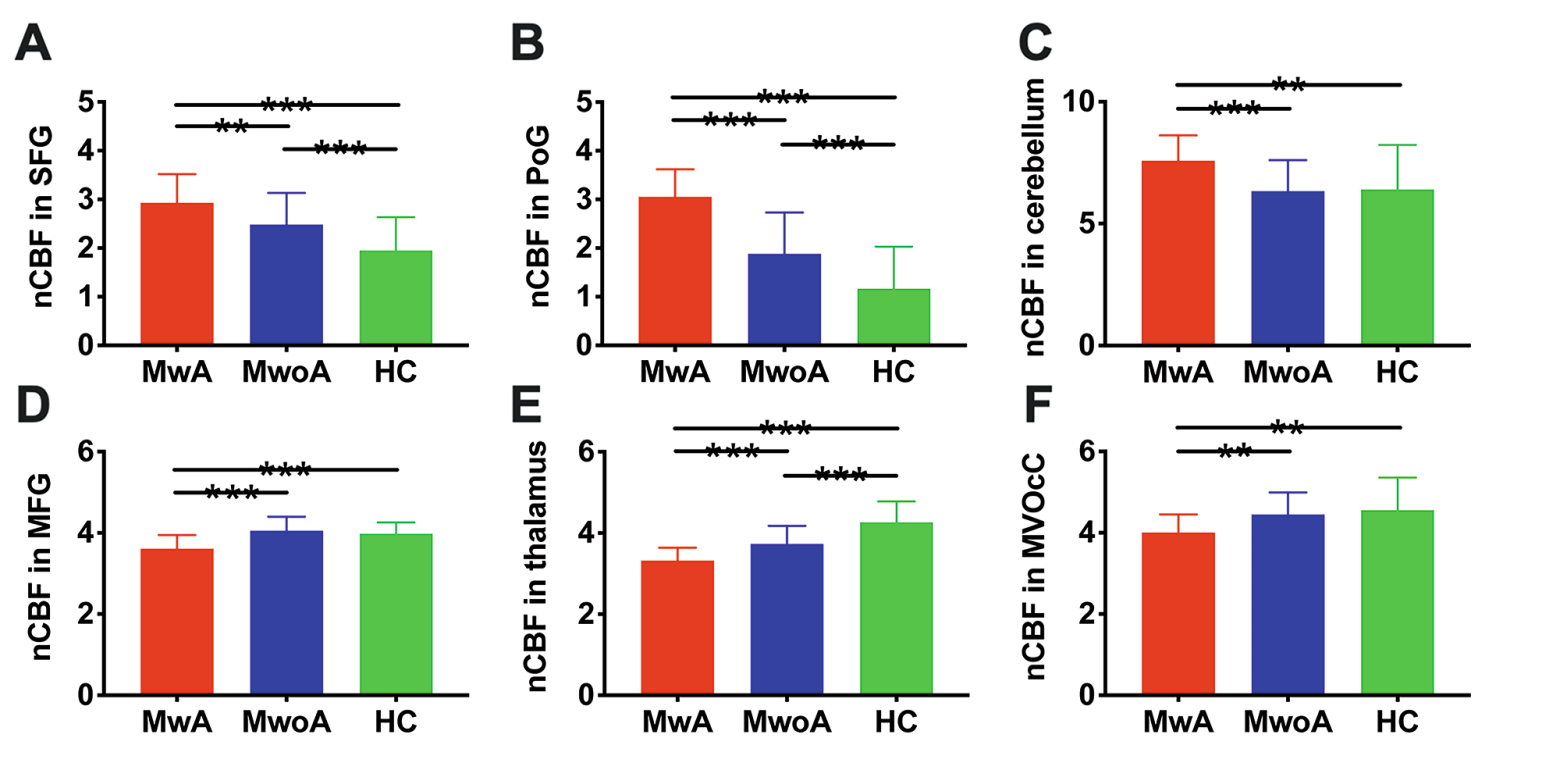
ROI-based comparison among MwA, MwoA and HC. The normalized CBF in six ROIs including superior frontal gyrus (SFG), postcentral gyrus (PoG), cerebellum, middle frontal gyrus (MFG), thalamus and medioventral occipital cortex (MVOcC) all showed significant differences between MwA and MwoA patients, and also between MwA and HC. Statistical significance is indicated by asterisks (***, p < 0.001; **, p < 0.01). HC: healthy controls, MwA: migraine patients with aura, MwoA: migraine patients without aura, nCBF: normalized cerebral blood flow

In comparison to MwoA, MwA exhibited significantly higher CBF levels in the bilateral superior frontal gyrus (SFG), bilateral postcentral gyrus (PoG) and cerebellum, and lower CBF levels in the bilateral middle frontal gyrus (MFG), thalamus and medioventral occipital cortex (MVOcC) (t > 3.0 and p < 0.05, FDR corrected at cluster level; Fig. 1). The further ROI-based comparisons showed significant differences in the mean normalized CBF value of these six ROIs between MwA and MwoA patients, MwoA patients and HC, and also between MwA patients and HC (all p values > 0.01, after FDR correction; Fig. 2). Comparing to HC, higher normalized CBF in SFG, PoG and cerebellum, as well as lower normalized CBF in MFG, thalamus and MVOcC were showed in MwA patients. Comparing to HC, higher normalized CBF in SFG and PoG, as well as lower normalized CBF in thalamus were showed in MwoA patients.

### Correlations between extracted CBF features and clinical scale assessment

**Fig. 3:**
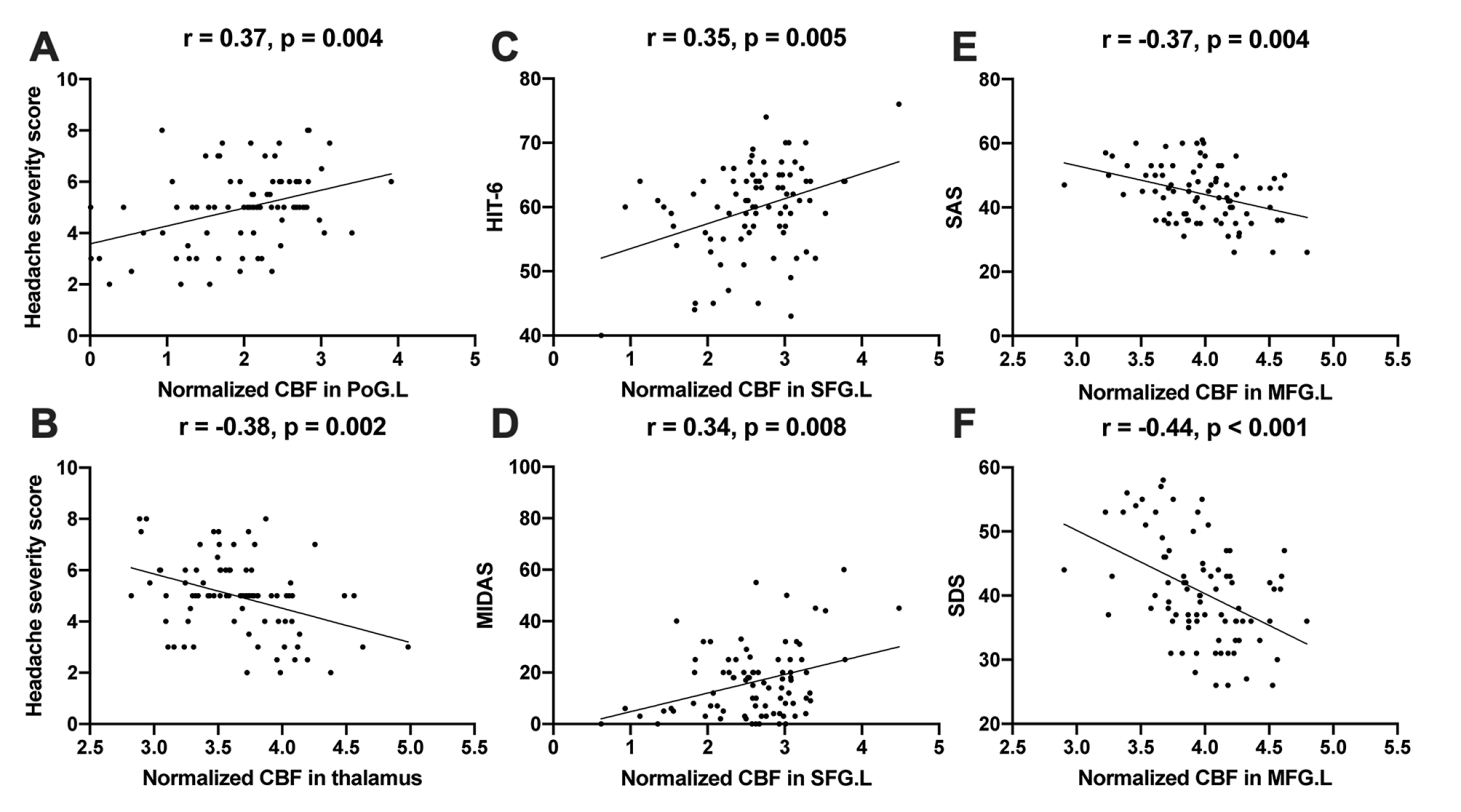
Relationship between the identified CBF features and clinical rating scales. There were significant correlations of normalized CBF in left postcentral gyrus (PoG.L) and thalamus with headache severity score, normalized CBF in left superior frontal gyrus (SFG.L) with HIT and MIDAS, normalized CBF in left middle frontal gyrus (MFG.L) with SAS and SDS among all patients (A-F, all p values < 0.01, after FDR correction). CBF: cerebral blood flow, HIT-6: Headache Impact Test-6, MIDAS: Migraine Disability Assessment Score, SAS: Self-Rating Anxiety Scale, SDS: Self-Rating Depression Scale

Several features were revealed to significantly correlated with clinical rating scales in all patients, including normalized CBF in left PoG and headache severity score (r = 0.37, p = 0.004 after FDR correction), normalized CBF in left SFG and HIT-6 (r = 0.35, p = 0.005) and MIDAS (r = 0.34, p = 0.008), normalized CBF in left MFG and SAS (r= -0.37, p = 0.004) and SDS (r = -0.44, p < 0.001) (Fig. 3). These correlations were all weak to moderate.

### The performance of machine learning–based models

**Fig. 4:**
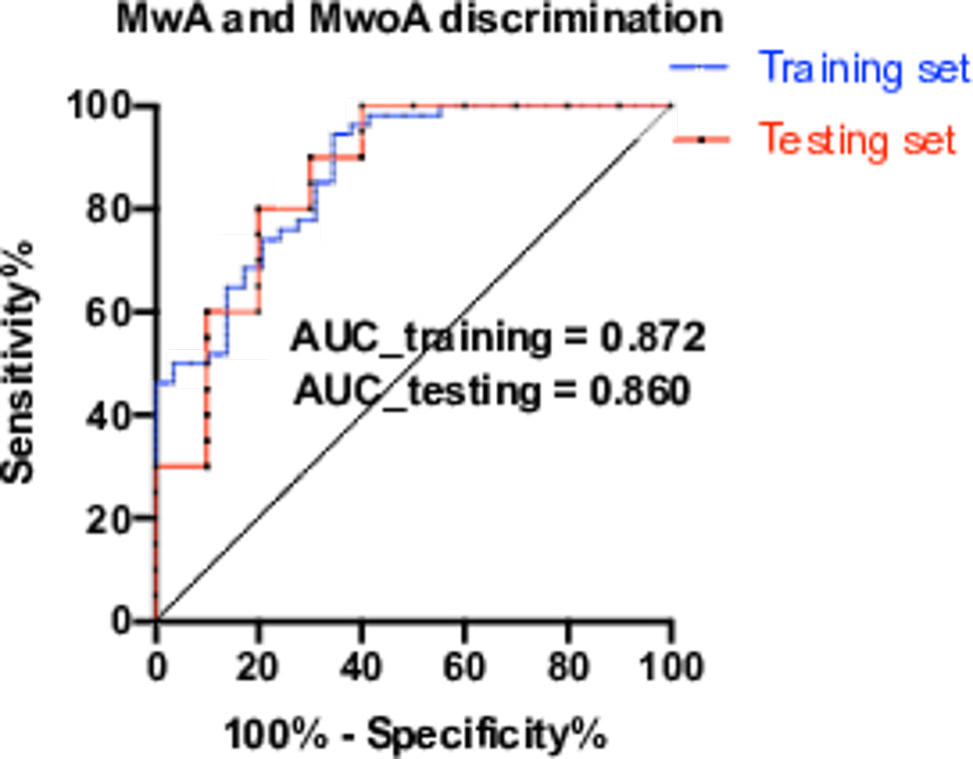
ROC curves were employed to evaluate the performance of the SVM model in the training and testing sets. The accuracy and area under curve of the SVM model in the training and testing sets were 84.3% and 0.872, 83.3% and 0.860 in discriminating patients with and without aura, respectively

Based on the six identified CBF features (normalized CBF in SFG, PoG, cerebellum, MFG, thalamus and MVOcC), the performance of machine learning–based models were listed in Table 2. Among them, the SVM models outperformed under 100 runs of five-fold cross validation. The accuracies and area under curve (AUC) values of the SVM model in the training and testing sets were 84.3% and 0.872, 83.3% and 0.860 in discriminating patients with and without aura, respectively (Fig. 4).

**Table 2:** Performance metrics from machine learning–based models

Machine learning-based models	AUC (%)	Accuracy (%)	Sensitivity (%)	Specificity (%)
SVM	84.1 ± 7.8	81.3 ± 6.2	87.6 ± 8.9	74.0 ± 6.9
KNN	67.2 ± 5.8	64.5 ± 7.0	70.9 ± 6.0	60.4 ± 4.7
RF	77.4 ± 6.1	74.2 ± 6.7	75.8 ± 6.3	72.1 ± 5.2
NB	64.2 ± 5.4	63.0 ± 4.8	68.3 ± 5.2	59.4 ± 4.1
LDA	76.3 ± 6.7	73.7 ± 5.2	77.0 ± 5.8	70.2 ± 7.1

AUC: area under curve, KNN: k-nearest neighbor, LDA: linear discriminant analysis, NB: naive bayes, RF: random forest, SVM: support vector machine

Values are represented as the mean ± standard deviation

## Discussion

Our study revealed that comparing to MwoA, MwA patients showed higher CBF in the bilateral SFG, bilateral PoG and cerebellum, while lower CBF in the bilateral MFG, thalamus and MVOcC. To our knowledge, this is the first time that a SVM model was applied to combine these brain blood flow features and achieved an accuracy of 83.3% to discriminate MwA from MwoA patients.

This study conducted voxel-wise analysis to extract blood flow features at a quantitative level that significantly contribute to subtyping migraine. Machine learning method has been considered as promising techniques that allow identifying potential imaging markers that could be used for diagnosis, treatment planning and disease progress monitoring of migraine [18, 19, 21, 22]. In a study of fMRI-based neural marker for MwoA, Tu et al. applied a recursive feature elimination (RFE) combined with SVM to select the functional connectivity features, as well as leave-one-out cross-validation (LOOCV) strategy to estimate the performance, they achieve an accuracy of 92.9% and area under curve of 0.97 to discriminate MwoA from HC [18]. The performance was quite promising and better than our results (the accuracies and AUC values in the training and testing sets were 84.3% and 0.872, 83.3% and 0.860 in discriminating MwA and MowA, respectively), but they did not have any results of the model performance in discriminating MwA and MwoA [18]. In Yang et al’s study, identifying fMRI features as low-frequency fluctuations (ALFF), regional homogeneity (ReHo), and regional functional correlation strength (RFCS) to discriminate MwoA and MwA with an inception module could reach accuracies at 89.77% ± 0.39%, 93.44% ± 0.20% and 96.13% ± 0.22%, respectively, with AUC of 0.99, which are higher than our results [21]. However, their sample sizes of MwoA and MwA were 21 and 15, which were relatively small. Moreover, both Tu et al. and Yang et al’s studies selected the feature derived from fMRI, which does not directly reflect brain blood perfusion situation as CBF does [18, 21]. Comparing to qualitative results that provide brain hyper- or hypo-perfusion, estimation of regional brain blood flow at quantitative level may provide direct index for clinician in discriminating migraine subtypes. With the advantage of sparing contrast agent, ASL sequence in migraine might be more recommended than dynamic contrast MR scan in estimating blood flow index. Importantly, the CBF imaging markers in our study achieved satisfying accuracies of 84.3% to discriminate MwA and MwoA in the training set by using the SVM model. We validated our model with an independent sample of migraine patients scanned in another MR system, with an accuracy of 83.3%. Together, it is suggested that the difference of normalized CBF between MwA and HC, as well as between MwA and MwoA we identified based on ASL could serve as imaging markers to subtype MwA and MwoA, and this finding is replicable across different coils, MR systems and sites.

The alternated normalized CBF in six brain regions overlaid important component of neuro-networks and circuits modulating migraine pain processing [23], and showed consistency with previous studies in differentiating MwA from MwoA, also MwoA from HC. In coherence to our results, previous fMRI results also demonstrated BOLD signal alterations, though reflecting indirect blood supplement variations, in frontal, occipital, thalamus and cerebellum in migraine patients [18, 24–26]. The involved frontal and occipital cortex may be corresponding to the aura related cortical spreading depolarization theory that the electric excitement initiating from the occipital lobe and spreading to the frontal lobes [23]. The alteration of normalized CBF occur to cortical regions (MFG, MVOcC, PoG, SFG) in our MwA group may responsible to their manifested symptoms, including visual/retinal aura (n = 22), sensory aura (n = 8) and literary aura (n = 4), motor aura (n = 1). The PoG perceives somatic sensation and plays critical role in the trigemino-thalamo-cortical pathway that has been demonstrated in the mechanism of migraine [27]. In the pathophysiology pathway, trigeminovascular neurons projecting to the somatosensory, motor, insular, parietal association, and visual cortices are responsible to the manifestation of migraine [27]. The MFG is demonstrated to be involved in modulation of pain [23]. The PoG and MFG are active during nociceptive processing and also are components of so called ‘pain matrix’, which is considered to integrate all sensory, affective, and cognitive responses to pain [23]. The SFG is reported to be associated with the executive functions. The deficit occurring in the SFG may affect the working memory function, verbal and spatial functions [28]. The visual area (MVOcC) is reported to correspond to the visual and retinal aura, which is considered as the characteristic manifestation of MwA [23, 27]. The significantly decreased normalized CBF in thalamus showed discriminative power to identify MwoA from HC may correspond to the photophobia that often manifested in MwoA [23, 29]. Photophobia could also manifest in MwA patients. Noseda et al. demonstrated that photophobia might be modulated through theretino-thalamo-cortical pathway in migraine [29]. Posterior thalamic nuclei contain a number of dura- and light-sensitive neurons that initially become active in migraine patients. These neurons also become sensitized, which can mediate the whole-body allodynia of migraine. The decreased normalized CBF in the thalamus of MwA corroborated photophobia phenomenon and gave the evidence of discrimination to identify MwA from HC in the present results [30]. Different from our results demonstrating an increased normalized CBF in SFG.L in MwoA, previous studies showed a decreased normalized CBF in MwoA [20, 31]. Meanwhile, previous studies demonstrated significantly increased or decreased CBF in MFG in MwoA [20, 31], but our results showed no significant difference between MwoA and HC (Fig. 3). The appearances of hyper- and hypo- perfusion brain areas of migraine patients in our study showed consistency to previous results, which demonstrated the co-existed increasing and decreasing perfusion in various brain areas and could even the same brain region in migraine patients [11, 25, 37, 38]. Combing our results with present studies, it comes to an agreement that there exists perfusion abnormality on ASL in migraine patients. In a pediatric migraine with aura mimicking stroke study, it is demonstrated that brain hypo-perfusion was followed by hyper-perfusion within 12 h of symptom onset [32]. Thus, the time courses may relate to the blood flow index obtained from ASL sequence scan.

The decreased blood flow in thalamus in our MwA and MwoA group may correspond to its role of modulation in pain process in migraine. The thalamus is an important structure of trigeminovascular nociceptive transmission-descending projections, the dysfunction of which is thought to contribute to triggering migraine attacks [23]. Thus, thalamic alteration at different perspectives were studied and reported in migraine patients. As a node of thalamocortical connection, alteration of thalamus in functional connectivity and structural connectivity were reported in chronic migraine, MwoA, migraine with complex neurological auras [18, 33, 34]. The volume alteration of thalamus and its sub-regions in migraine were controversial, most recent study reported no volume alteration in thalamus in female MwA patients [35–37]. Reduced total N-acetyl-aspartate and total creatine in thalamus of chronic migraine patients were reported previously [38]. In a molecular imaging study, increased [11 C] PBR28 was detected in thalamus indicating neural neuroinflammation in MwA [39]. The decreased CBF and metabolism index combining increased inflammatory activity may all contribute to the dysfunction of thalamus leading to flaw modulation of trigeminocervical complex, which results in intra- and extra-cranial somatosensory information improperly processed, and ultimately perceived by migraine patients.

The correlations between alteration of normalized CBF and the clinical assessment in our study implied a migraine disease progress pattern and clinical manifestation. Four CBF alteration markers showed correlations to headache severity, emotional state and migraine disability assessment in migraine. The positive correlation between HIT-6/MIDAS and normalized CBF in left SFG demonstrated the increased blood flow may represent the exciting cortex in superior frontal gyrus that may affect headache modulation leading to the decrease of life quality. The negative correlation between SAS/SDS and normalized CBF in left MFG implies increased blood flow in frontal lobe would decrease emotional influence derived from the migraine. The hyper-perfusion in PoG and hypo-perfusion in thalamus worsen the headache and release the headache respectively which may relate to the modulation role of the brain regions and the time course of the migraine onset [32, 33]. The migraine-stroke connection progress may involve microemboli or other factors causing regional CBF variations lead to cortical spreading depolarization and then trigger headache, which proceed in a short time course during headache attack onset phase and hard to catch [40]. Present results with associations of clinical manifestations and the alternated CBF in our migraine group demonstrated that the potential imaging markers also provide important information at headache interictal stable phase.

There existed some limitations in this study. First, we did not recruit migraine patients with aura symptom onset or short term after aura symptom onset, which leaded to our results lack of time course value in migraine aura-stroke mimic connections. Second, we did not classify our MwA group into migraine with brainstem aura, or hemiplegic migraine and retinal migraine to investigate the possible pathophysiological mechanism underlying particular subtypes of MwA. Third, the MR scanning and the clinic assessment were conducted during the migraine ictal-phase, which might be short of information of real-time headache attack. Last, to avoid overfitting, we conducted the feature selection before the model construction. Thus we used only identified CBF differences between MwA and MwoA patients as features to build models to discriminate MwA from MwoA patients. In consideration of the sample size (88 in the training sample) and feature number (N = 6), we chose to construct the SVM models with the gaussian kernel to differentiate MwA and MwoA under 100 runs of five-fold cross validation. The cross validation and external validation could guarantee the acceptable robustness and generalization of the final model. However, our model still need to be further evaluated and confirmed with more patients from multiple cohorts. More detailed subtypes and time course information of migraine to describe disease progress pattern and stroke incidence rate are needed in further study to validate our CBF markers to assist in optimal precision medicine and prognosis of migraine patients.

## Conclusion

In summary, present work confirmed the alterations of regional cerebral blood flow in migraine with aura, compared to those without aura and healthy controls. It’s noteworthy that these potential imaging markers might help better understand migraine-stroke connection mechanisms and may guide patient-specific decision-making.

## Data Availability

All data and materials generated in this study are available upon request.

## List of abbreviations

ASL: Arterial spin labeling.
AUC: Area under curve.
BOLD: Blood-oxygen-level-dependent.
CBF: Cerebral blood flow.
DCE: Dynamic contrast-enhanced.
FD: Frame-wise displacement.
FDR: False discovery rate.
fMRI: Functional MRI.
HC: Healthy control subjects.
HIT-6: Headache impact test-6.
ICHD: International classification of headache disorders.
MFG: Middle frontal gyrus.
MIDAS: Migraine disability assessment score:MoCA:Montreal cognitive assessment:MVOcC:Medioventral occipital cortex.
MwA: Migraine with aura.
MwoA: Migraine without aura.
PoG: Postcentral gyrus.
ROC: Receiver operating characteristic.
ROI: Region of interest.
SAS: Self-rating anxiety scale.
SD: Standard deviation.
SDS: Self-rating depression scale.
SFG: Superior frontal gyrus.
SVM: Support vector machine.
